# Distal and Proximal Religiosity as Protective Factors for Adolescent and Emerging Adult Alcohol Use

**DOI:** 10.3390/rel6020365

**Published:** 2015-04-02

**Authors:** Michelle V. Porche, Lisa R. Fortuna, Amy Wachholtz, Rosalie Torres Stone

**Affiliations:** 1 Wellesley Centers for Women, Wellesley College, 106 Central St., Cheever House, Wellesley, MA 02481, USA; 2 Boston Medical Center, Dowling Building, 9th Floor, Boston, MA 02118, USA; 3 UMASS Memorial Medical Center, Department of Psychiatry, University of Massachusetts Medical School, 55 Lake Avenue North, Worcester, MA 01655, USA; 4 Systems & Psychosocial Advances Research Center, University of Massachusetts Medical School, Department of Psychiatry, 55 Lake Avenue North, Worcester, MA 01655, USA; 5 Department of Sociology, Clark University, 950 Main Street, Worcester, MA 01610, USA

**Keywords:** addiction, adolescence, alcohol use, childhood adversity, emerging adulthood, religion, spirituality

## Abstract

Data from emerging adults (ages 18–29, *N* = 900) in the National Comorbidity Survey Replication Study was used to examine the influence of childhood and emerging adult religiosity and religious-based decision-making, and childhood adversity, on alcohol use. Childhood religiosity was protective against early alcohol use and progression to later abuse or dependence, but did not significantly offset the influence of childhood adversity on early patterns of heavy drinking in adjusted logistic regression models. Religiosity in emerging adulthood was negatively associated with alcohol use disorders. Protective associations for religiosity varied by gender, ethnicity and childhood adversity histories. Higher religiosity may be protective against early onset alcohol use and later development of alcohol problems, thus, should be considered in prevention programming for youth, particularly in faith-based settings. Mental health providers should allow for integration of clients’ religiosity and spirituality beliefs and practices in treatment settings if clients indicate such interest.

## 1. Introduction

Experimentation is central to development as adolescents move into emerging adulthood, the developmental period from the late teens into the 20s [1], when there is also increased risk for under-age and binge drinking [2], alcohol use disorders, and impaired driving [3–5]. It is a period of life when young people continue to explore and mature their understanding of religion and spirituality [6]. Religiosity and spirituality are resources that can lessen risk behaviors and enhance positive outcomes [6–8]. Religiosity includes practices and activities that are related to a religious tradition, institution or community, e.g., devotional practices within a community or regular church attendance; or belief, at least in part, to a set of religious doctrines or values [9]. Religiosity can contribute to health decision making related to alcohol and other substance use [9], by reducing alcohol use and risk behaviors leading to alcohol use disorders [10]. In this paper, we examine associations of religious participation and religious-based decision-making on early onset alcohol use, as well as later prevalence of alcohol use disorders, in a diverse and nationally representative sample of emerging adults.

### 1.1. Religiosity in Adolescents and Emerging Adults

Emerging adulthood is a time of transition from the externalized behaviors recommended by one’s religion (e.g., attendance at services) to the internalization of concepts of morality, religiously based decision-making, and behavior [11]. The process of developing and internalizing conceptual belief models shape an individual’s world view and, consequently, behavior and mental health. There is often an intense period of reflection of existential questions such as the simultaneous existence of both good and bad in the world; reconciling disconnects between self, personal beliefs, and the external environment and behaviors of others [12]. Among emerging adults attending college, this process is related to the coursework that students take, the philosophical questions they ask, and their initial concept of a higher power [12]. However, little is known about the impact of these struggles on emerging adults who are not attending college, or the impact that religiosity has on mental health and health behaviors among a more ethnically diverse group of emerging adults.

Religious beliefs and practices can potentially affect alcohol use through influencing individual and group practices within a socio-environmental context. For example, church communities that speak actively against alcohol and substance use, support family networks and prosocial and academic endeavors could provide the doctrine and environment that protects against alcohol abuse and promotes religiously based decision-making [13,14].

Religiosity may be particularly protective during the transition period from adolescence to emerging adulthood. A review by Rew and Wong [15], exploring the impact of religiosity on health attitudes and behaviors among this age group found that religiosity had a positive impact on health behaviors and alcohol use in 84% of the studies reviewed. Newcomb *et al.* [16] identified religious commitment as a factor that significantly decreased alcohol use among high school students. Using nationally representative data from 20 years of Monitoring the Future surveys of adolescents, Wallace and Forman [17] identified a consistent negative association across survey years in that adolescents who scored higher on religious commitment were less likely to engage in drinking behaviors. Further research suggests that it is not only external commitment, but the internalized religious beliefs of adolescents that have the greatest influence on recent drinking experiences [18]. Each of these studies suggests that if adolescents make the personal choice to engage in religious or spiritual activities, they are more likely to internalize healthy behavior and decision making into their adulthood.

Wong *et al.* [19] conducted a systematic review of the 20 studies published between 1998 and 2004 that explored the role of religious variables on mental health in older adolescents and emerging adults. Institutional components of religiosity such as church attendance were significantly linked to positive mental health, whereas personal devotion had a limited impact on mental health. This is in direct contrast to a meta-analysis among established adults by Hackney and Sanders [20], in which the key aspect of religiosity related to positive mental health is personal devotion, with institutional components being the weakest predictor. While this review is limited, it does provide some evidence that specific aspects of religion may have differential importance for emerging adults compared to established adults [19]. Perhaps for adolescents and emerging adults the influence of institutional components of religion, church attendance, and church support play an important but early role in behaviors, and later this religiosity is internalized to influence decision-making.

### 1.2. Early Onset of Alcohol Use and Dependence for Adolescents and Emerging Adults

The detrimental effects of early onset of alcohol use (loosely defined by various researchers from ages 14 to 16) are recognized as an important public health issue [21,22]. Research has found that youth who have their first drink at age 14 are four times more likely to develop alcohol dependence compared with emerging adults whose initial drinking experience is age 20 or older [23]. Early onset of alcohol use is associated with progression to more severe alcohol abuse, greater likelihood of subsequent development of alcohol dependence and the use of other drugs especially among the youngest initiators [24–26]. Early onset is also associated with other problem behaviors, such as academic failure [27], dropping out of school [28–30], and difficulty sustaining employment [31]. Early onset of alcohol use is also associated with early adolescent sexual activity [32], sexual risk-taking behavior [33], and related sexually transmitted diseases (STD) and HIV exposure [34]. Commonly recognized risk factors for early onset of alcohol use include peer influence, ineffective parenting, and disadvantaged social context [24,35].

Dishion *et al.* [24] suggest that a combination of peer and family influences interact in a manner to represent a “childhood risk” for substance abuse; their study of adolescent males found peak timing for alcohol initiation at grade nine. In a longitudinal study of emerging adults from ages 18 to 25 [4], highest levels of alcohol consumption was at age 18, with decline over time and leveling off at around 24 years of age. However, being male, as well as having more baseline dependence symptoms and greater alcohol and legal problem severity are associated with greater consumption and binge drinking [4]. In addition, greater levels of binge drinking are associated with less education, earlier age of first use, and a larger social network of heavy drinkers [4]. There are multiple, individual, familial and social environmental factors, including early adversity, which can have an impact on the development of alcohol use disorders [36].

### 1.3. Theoretical Model

Bandura’s social cognitive learning theory provides a useful framework in which to examine the influence of religiosity on alcohol use behaviors among emerging adults [37,38]. The emphasis on agency and self-regulation are key to understanding mechanisms related to risk and resilience, relapse and recovery. The social learning model dovetails with Pargament’s empirically validated model on religion (and spirituality) that describes a psychologically distal/proximal framework [39,40]. The distal domain comprises external behaviors such as attendance at religious functions, or reading religious materials. The proximal domain measures the function, or the internal representation of the external behaviors, including internalizing beliefs, meaning making, and religious coping (use of religious beliefs or spirituality in decision making and managing life challenges). As emerging adults develop, the emphasis changes from primarily distal/external behaviors to the inclusion of more proximal/internal beliefs [15].

This study combines these two frameworks into a developmentally appropriate model that examines aspects of both distal (*i.e.*, participation with a faith community and church attendance) and proximal functions of religion (*i.e.*, religious-based decision making) to identify correlates of drinking behaviors for emerging adults. Social interactions can also present ecologically proximal or distal opportunities for social learning of risky or protective behaviors, and may do so in cumulative or multiplicative ways [28,41]. Family, peers, school, and neighborhood comprise the more proximal influence for risk behaviors, especially during childhood and adolescence [42–44]. However, the influence of parents becomes more distal and the influence of peers more proximal from adolescence into early adulthood [45]. Parental monitoring operates within a constellation of family and neighborhood systems [5,46]. In the wider macrosystem societal dimensions of culture, including religious domains, and tradition/cultural values interact with more proximal influences to affect alcohol use [47–50]. For emerging adults, risk factors are dynamic and multi-dimensional, and dependent on current and changing social contexts [51]. Thus, a developmental approach is required that considers both present and past social contexts, including family supports and shared values (religiosity) in childhood [43,52–54] in modeling behaviors.

## 2. Methods

The primary aim of this study is to test dimensions of religiosity and religious-based decision making in childhood and emerging adulthood as potential diverters of early onset alcohol use and in the prevention of alcohol use disorders. We test these religious dimensions as potential protective correlates of early age of alcohol use (reported age 15 or younger) and/or alcohol use disorders along with other dimensions of family life, cultural affiliation, and childhood environmental stressors and adversity in a nationally representative sample of young adults.

### 2.1. Sample

Secondary data analysis was conducted with the National Comorbidity Survey Replication (NCS-R) [55] to examine the multi-dimensional role of religiosity in emerging adults. This dataset is of three nationally representative household surveys that comprise the Combined Psychiatric Epidemiological Surveys (CPES), one of the largest psychiatric epidemiological studies of ethnic racial minorities and non-Latino Whites. The National Comorbidity Survey Replication (NCS-R) subset is used alone because it includes a racially and ethnically diverse sample and contains key religiosity variables of interest as well as information about childhood adversity. Other components of CPES include the National Survey of American Life (NSAL) [56] targeting a Black sample (African Americans and Afro-Caribbeans), and the National Latino and Asian American Study (NLAAS) [57] include some overlapping questions of the NCS-R but, for this study, lack variables of interest related to childhood adversity. This data is de-identified and publicly available, thus exemptions for human subjects research apply.

The CPES surveys were developed under the sponsorship of the National Institute of Mental Health (NIMH), and the data collection was conducted by the Survey Research Center (SRC) of the Institute for Social Research at the University of Michigan, from early 2001 through to the end of 2003. In-person interviews were conducted unless telephone interviews were requested or travel was prohibitive for interviewers. All respondents completed core protocol and screening questions (approximately 2.5 h); additional sessions may have been necessary to complete follow up related to screening. The protocols were translated and bilingual interviewers were trained so that non-native English speaking respondents could answer in their native languages. This non-institutionalized community sample excluded incarcerated individuals or those residing in contained mental health facilities. More detailed information of the sample design and weighting is described by Heeringa *et al.* [58]. The full CPES sample includes data from 20,013 adults, ages 18 and older, who participated in face-to-face structured interviews. The subsample for this analysis is limited to the emerging adults ages 18 to 29 (*n* = 900) from the NCS-R subset of the CPES, which is a probability sample of the United States. The sample was evenly matched by gender (50.10% males and 49.90% females, weighted).

### 2.2. Measures

#### Early Onset of Alcohol Use

The dependent variable is derived from a question that asked the age when the participant first drank alcohol. The outcome, early onset of alcohol use, was coded as 1 if respondents indicated that the first drink occurred at age 15 or younger, and coded as 0 if age 16 or older.

#### Early Regular Alcohol Use

The dependent variable is derived from a question that asked the age when the participant first “drank 12 or more drinks per year”. The outcome, early regular alcohol use, was coded as 1 if respondents indicated that they had 12 or more drinks per year at age 15 or younger, and coded as 0 if age 16 or older.

#### Any Lifetime Alcohol Use Disorder

The WHO CIDI, a comprehensive structured interview designed for clinical and research purposes, asks about mental disorder symptoms according to the definitions and criteria of International Diagnostic Codes of Disorders and the DSM-IV. This includes lifetime disorders and age of onset (past-12-month criteria symptoms were also collected but are beyond the scope of this analysis). The comprehensive 47-question Alcohol Use section was administered by investigators who had undergone over 30 h of training to ensure reliable assessment of full diagnostic criteria. Questions in this section also document history, severity, burden, service use, medication, and treatment. Responses are then compiled to provide the diagnosis that is included in the public use dataset. A dichotomous variable was coded as 1 if a DSM-IV diagnosis of alcohol abuse or alcohol dependence was indicated by responses on the CIDI; otherwise alcohol use disorder was coded as 0.

#### Childhood Religiosity

Respondents were asked to assess “How important was religion in your life when you were growing up” on a 4-point scale (1 = *not at all important*; 4 = *very important*).

#### Emerging Adult Religiosity

Frequent Church Attendance. Respondents were asked, “How often do you usually attend religious services?” This 5-point scale was recoded into a dichotomous variable where *more than once a week, about once a week, and one to three times a month* were coded as 1, while *less than once a month* and *never* were coded as 0.

#### Religious Beliefs Guiding Decision-Making

Respondents were asked, “When you have decisions to make in your daily life, how often do you think about what your religious or spiritual beliefs suggest you should do—often, sometimes, rarely, or never?” This was recoded into a dichotomous variable where responses of *often* or *sometimes* were coded as 1 and responses of *rarely* or *never* were coded as 0. Because the item response “sometimes” could have a wider interpretation than we assume for this analysis (e.g., someone might respond “sometimes” for rare instances of this kind of decision-making) we also ran a sensitively analysis using the response “often” in comparison to all other responses.

#### Religious Denomination Affiliation

Respondents were asked to identity their religious preference and allowed to name up to three denominations or preferences (or indicate none). For analytic purposes we report on responses to first preference only. From the available categories, a series of dummy variables were created (CPES identifiers in italics) for Baptist (*All Types*), Catholic (*Roman Catholic*, *Other Catholic, Catholic Denomination Not Mentioned*), Protestant (*Protestantism/Protestant*, *Other Protestant*), Other Christian (*Lutheran*, *Methodist*, *Pentecostal*, *Presbyterian*), Other Religion (*Jewish, Hindu*, *and Muslim affiliations but not specifically identified because of small cell size*), and No Religious Preference. No one in the emerging adult sample was identified in the Agnostic or Atheist category.

#### Childhood Adversity

A series of questions regarding exposure to early adversity were asked including (1) if the respondent’s *family received government assistance for six months or more during the respondent’s childhood or adolescence* (1 = *yes*, 0 = *no*); (2) if *while growing up the mother or maternal guardian had a problem with alcohol or drugs* (1 = *yes*, 0 = *no*); (3) if *while growing up the father or paternal guardian had a problem with alcohol or drugs* (1 = *yes*, 0 = *no*); (4) if *while growing up the mother or maternal guardian had periods of sadness for two weeks or more* (1 = *yes*, 0 = *no*); (5) whether they were *frequently left unsupervised at too early an age in childhood* (recoded to a dichotomous variable 1 = *often* or *sometimes*, 0 = *rarely* or *never*); (6) whether they *frequently went hungry or parents didn’t fix meals in childhood* (recoded to a dichotomous variable 1 = *often* or *sometimes*, 0 = *rarely* or *never*).

#### Child Lived with Both Biological Parents

Respondents were asked to report whether they “lived with both biological parents until age 16”; this was coded as 1 if *yes* and 0 if *no*.

#### Control Variables

Gender was coded as 1 for *male* and 0 for *female*. Race/ethnicity was coded into four dummy variables: Asian, Black, Latino, and White (which was the reference group for the regression analyses). Education level was reported as the number of years of formal education completed (e.g., 12 years for high school graduate, 16 years for college graduate, 17 or more years for graduate study).

### 2.3. Analysis

Descriptive and inferential analyses were conducted using SAS 9.2 (SAS Institute, Inc., Cary, NC, USA). All analyses were weighted in order to account for the survey sampling design including the intentional over sampling of some subgroups, thus results presented are national estimates. First, we estimated the weighted prevalence of early onset of alcohol use, early onset of regular drinking, and alcohol use disorders for a sample of US born and immigrant respondents ages 18 to 29. Significance tests for group differences were conducted using a Rao-Scott chi-square statistic for contingency tables with survey data [59,60]. We also examined correlates of alcohol use and disorders in logistic regression models including religiosity measures, childhood adversity, and demographic variables. For Models 1 and 2, the variables were entered in the following conceptually distinct blocks: Demographics, Childhood Adversity and Protective Factors, and Religiosity variables. For Model 3, the same blocks were used, followed by the addition of Early Regular Drinker, and lastly, the interaction terms.

Missing values were multiply imputed using the PROC MI procedure in SAS [61,62]. Regression models were adjusted for sampling design through a first-order Taylor series approximation, and significance tests were performed using design-adjusted Wald tests. We report odds ratios and 95% confidence intervals. Based on chi-square results, additional tests for the moderating effect of gender were conducted by creating interaction terms with importance of religion as a child, frequent church attendance as an adult, and religious beliefs guiding decisions. The ODDSRATIO statement for SAS, which provides customized odds ratio output specific to levels of categorical and continuous variables, was used to output stratified odds ratios for the interaction terms.

## 3. Results

### 3.1. Descriptive Results

The majority of the sample (53%) reported trying their first drink at age 15 or younger. Almost a fourth of the sample (22%) reported early regular drinking (12 or more drinks in a year) starting at age 15 or younger. The lifetime prevalence rate for any alcohol use disorder (alcohol abuse or alcohol dependence) was 14%. Although there was no gender or racial difference for early onset of drinking, there were significant gender and racial differences for drinking patterns (see Table 1 for descriptive results). Compared to young women, a greater percentage of young men reported drinking regularly at an earlier age (25% *vs.* 19%) and young men’s rate for any alcohol use disorder was double that of young women (19% *vs.* 10%). There were also significant differences in alcohol use and religiosity by race/ethnicity (Table 2). White respondents included the highest percentage of early regular drinkers (22%) and Asians the lowest (6%).

Religion played a prominent role in the childhoods of most of the sample; 35% rated it *very important* and 39% rated it *somewhat important*, while 20% reported it was *not very important* and 6% rated it as *not at all important*. These patterns were also reflected in emerging adults’ reports of current religiosity: 63% reported that religious beliefs guided their decision-making *often* or *sometimes*, and 43% reported attending church regularly (once a month or more). Weighted correlation coefficients showed that importance of religion as a child was moderately correlated with adult church attendance (*r* = 0.35, *p* < 0.0001) and a weaker correlation with religious beliefs guiding decisions as an adult (*r* = 0.17, *p* < 0.0001). Religious preferences were reported (as per conventions used by the NCS-R) in the following order: Catholic (29%), Protestant (19%), Baptist (17%), Lutheran (6%), Methodist (6%), Presbyterian (3%), Pentecostal (2%), other preference (9%; although Jewish, Hindu, and Muslim affiliations are included, only aggregated total is available), and no preference (9%). Chi-square tests found no significant associations between any of the denominations identified above and the three outcomes (e.g., Baptists *vs.* all others, *etc.*). No Religious Preference was the only significant category, with respondents who reported no preference being more likely to report early onset of drinking (weighted 78% *vs.* 49%, F = 12.71, *p* < 0.001) and early regular drinking (20% *vs.* 41%, F = 14.99, *p* < 0.001) compared to those who reported affiliation with a particular denomination. Given the narrow results using denomination categories and the aim of building parsimonious models, we did not include these variables in our logistic regressions. Compared to young men, a greater percentage of young women endorsed the importance of childhood religiosity, reported frequent church attendance as adults, and used religious beliefs to guide decision-making. Group comparisons showed that White respondents claimed childhood religiosity as important less often (73%) and that religious beliefs guided their decision-making less often (62%) than Asian, Black, and Latino groups (see Table 2).

### 3.2. Logistic Regression

Childhood religiosity was associated with reduced likelihood of early onset drinking (first drink at age 15 or younger; Odds Ratio = 0.56) controlling for demographic covariates (Table 3, Model A). Living with both parents was also associated with reduced odds of early onset drinking (OR = 0.54) compared to those who did not live with both parents, whereas retrospective reports of paternal substance use (OR = 1.89) and maternal depression (OR = 1.79) were associated with greater likelihood of early onset drinking compared to respondents without these parental conditions.

However, childhood religiosity, which was negatively related to being an early regular drinker in an unadjusted model (OR = 0.57 [0.34,0.95] not shown), did not significantly offset the types of childhood adversity associated with early onset of regular use (12 drinks or more per year at age 15 or younger, when included in the adjusted model; Table 3, Model B). Males were 1.61 times more likely to report early drinking than females (OR = 1.61). Respondents who reported maternal substance use were 2.15 times more likely to report early drinking (OR = 2.15) compared to those without maternal substance use. Those whose mothers had depression were almost twice as likely to have early onset drinking (OR = 1.95) compared to peers without reported maternal depression, and those often left unsupervised as young children were over three times as likely (OR = 3.53) to have early onset drinking compared to supervised youth. Asian and Black emerging adults were less likely have early onset of regular drinking (OR = 0.29 and 0.27, respectively) compared to White counterparts. Going hungry as a child was associated with delayed onset of regular drinking (OR = 0.13) compared to peers who did not report this experience.

For emerging adults, the importance of religiosity as a child had a significant negative association with any alcohol abuse or dependence in an unadjusted model (OR = 0.63 [0.40,0.95] not shown) but was not significant in the adjusted model. Frequent church attendance was associated with reduced odds of any lifetime alcohol abuse or dependence (OR = 0.21) compared to those who attended less than once a month, however gender moderated the effect of frequent church attendance as well as childhood importance of religion (Table 3, Model C). Interpretation tests that provided stratified odds ratios showed this effect was significant for females but not for males, as odds of alcohol abuse or dependence were reduced for females who rated religion as important during childhood (OR = 0.46 [0.25,0.86]) or who reported more frequent church attendance as an adult (OR = 0.34 [0.17,0.67]) compared to those who did not. Religious beliefs guiding decision-making was not significantly related to abuse or dependence (either as coded *often* or *sometimes*, or as coded as *often* in a follow up sensitively analysis; not shown). Risk for alcohol abuse or dependence was over two times higher for those who reported maternal (OR = 2.87) or paternal (OR = 2.27) substance use, compared those who did not report parental substance use; Black emerging adults showed reduced odds (OR = 0.32) compared to White peers. The strongest association for lifetime alcohol abuse or dependence was early onset of regular drinking (OR = 7.25), with those who reported early onset being 7.25 times more likely to have any lifetime alcohol abuse or dependence compared to peers with later onset of drinking.

For all three adjusted models, the family being on welfare for six months or more was not significant when included with other variables that may also reflect poverty conditions, including maternal depression, lack of supervision, and food insecurity. In unadjusted models (not shown) welfare status was associated with increased odds of early onset of drinking (OR = 2.42 [1.25,4.68]), early regular drinking (OR = 2.14 [1.19,3.85]), and any lifetime alcohol abuse or dependence (OR = 2.43 [1.21,4.87]).

## 4. Conclusions

In our final adjusted regression models, religiosity in childhood (rating of importance of religion in childhood) was found to be negatively associated with first use of alcohol but not with early regular use, nor with lifetime abuse or dependence. In contrast, frequency of adult church attendance was negatively associated with meeting criteria for alcohol abuse or dependence. Given that childhood and adult religiosity variables were correlated, and the fact that simple bivariate regression tests showed that this childhood variable was negatively associated with a history of alcohol abuse or dependence, it may be that childhood religiosity has an influence beyond childhood that was not captured with our available data. Thus, longitudinal research is necessary to fully understand whether, to what degree, and under what conditions religious beliefs and practices in childhood may have a continued influence into adulthood so as to protect against alcohol problems across the lifespan. Our results support other research findings, such as those from the Add Health data describing distal (behaviors and attitudes) and proximal (internalized beliefs) religiosity as negatively associated with alcohol use frequency and quantity for young adults [63]. Extending that work, our study also examined multiple measures of religiosity and demonstrated similar relationships in a community sample of emerging adults.

### 4.1. Gender

Over half of sample participants tried alcohol for the first time at age 15 or younger despite the legal drinking age of 21. Although girls were as likely as boys to engage in early experimentation with alcohol, boys were more likely to start drinking regularly and young men were at greater risk for alcohol use disorders. An important finding in the current study is the differential relationship between childhood religiosity, adult church attendance and alcohol use disorder by gender in that high levels of religiosity was associated with reduced risk of alcohol abuse and dependence in females. Steinman and colleagues [64] found racial and gender variations in the dose-response of religious activity such that for 12th grade White males in a metropolitan setting, higher frequencies of engagement in religious activities were protective whereas occasional participation was related to more frequent alcohol use. This national study found these patterns of associations only for females. Existing research suggests that heavy drinking is part of masculine socialization and identity [65,66] and that religiosity is equated with the feminine rather than masculine [67,68]. The results of current study raise the question of whether other social circumstances may differ by gender and whether these might be associated with a differential influence of religiosity for boys. Overall, the literature shows that males are more vulnerable to alcoholism in the context of adverse family situations, antisocial fathers, and aversive environments [36]. But it is unclear how these factors interact with religiosity patterns in families, as we found that parental abuse continues to pose a risk for alcohol problems even in the context of high religiosity.

### 4.2. Ethnic Minorities and Alcohol Use

Results showed that prevalence rates for early regular drinking and alcohol use disorders were lowest for Asian and Black emerging adults. National longitudinal data from the Add Health Study shows the Whites have the highest rates of alcohol use from adolescent through emerging adulthood, with significantly lower rates for African American peers over time [69]. Previous research has found that African American youth are more likely to abstain from alcohol and less likely to develop alcohol problems as compared to their White counterparts and that religiosity appears to influence these lower rates among African Americans [70]. Our results, showing Whites having lower religiosity as children, were consistent with trends documented through analysis of three national data sets (Monitoring the Future 1996, Survey of Adolescent Health 1995, and The Survey of Parents and Youth, 1998) that found African American youth had more frequent church attendance and greater youth group participation compared to White peers [71]. Research on the influence of specific affiliations [72] suggest that denominations that are conservative in regards to alcohol use, and differentiate themselves from more permissive cultural norms, are also more protective against adolescent alcohol use. Black churches are important examples of this and may explain lower risk for African American youth. Although we did not find significant negative associations with alcohol outcomes for any one denomination, we did see that in general having any denominational preference was related to lower likelihood of alcohol outcomes compared to those having no religious denominational preference. While naming a preference may reflect a connection with a particular faith, across race and ethnicity, the dataset lacks information about depth of commitment and practice that would allow for deeper exploration of potential influence on alcohol use.

On the other hand, the rates we found for Latino alcohol use were more similar to White counterparts. Canino *et al.* [73] found that the rate of lifetime alcohol disorder among Latinos in the U.S. (in a National sample with an age range of 18 to 65 years and up), is close to 17%, which is similar to our findings. However, they also found that rates of alcohol abuse and/or dependence vary by nativity with a lifetime alcohol disorder rate of 9.7% among immigrant Latinos as compared to a rate of 27% among U.S. born Latinos. Several factors, including the acceptability of social drinking, may influence the prevalence of alcohol use disorders among ethnic minorities.

It is important to consider how religiosity may also interact with social and cultural expectations regarding adulthood across race and ethnicity. In one study, Arnett [74] found that although conceptions of adult status in emerging adults are similar across racial ethnic groups (e.g., becoming independent and self-sufficient) they differ in distinct ways by race, ethnicity, and socioeconomic status. African Americans, Latinos and Asian Americans, persons with relatively low socioeconomic status, and persons whose families had been in the U.S. relatively fewer generations were more likely than White individuals to support the criteria on the norm compliance subscales particularly “avoid becoming drunk” as a marker for adulthood. This greater concern among racial and ethnic minorities for the opinion of others may influence risky behavior such as alcohol use in this period of the life course. Although most studies have been conducted on White youth, there is some evidence that religiosity and spirituality serve as protective factors for drug and alcohol use among all racial and ethnic minorities [75,76] controlling for religious affiliation [77]. For instance, a study of predominantly Latino adolescents found that spirituality protected against marijuana and hard drug use [76]. In another study with a sample of Latino eighth-graders, Wallace [78] found that attendance at religious services was inversely related to drug use. There is some evidence, however, that the protective effect of religiosity and spirituality on alcohol use is not uniform within a single pan-ethnic minority group. For example, in a study of Latino youth, Latino gang members’ attendance at religious services did not protect against drug use as it did for Latino non-gang members [75]. Thus far there is limited understanding about how peer religiosity may be protective against alcohol abuse patterns [79] in the face of the strong influence that peer social context of drinking has in general [14].

Our study supports that higher religiosity in childhood and emerging adulthood as defined as more church attendance in these periods of life may be protective against early onset alcohol use and later development of alcohol problems. Religiosity is one of many factors that can influence alcohol use but the fact that it is associated with decreased risk in emerging adulthood is noteworthy for development of potential interventions. Significant correlations found between childhood religiosity and adult practices and beliefs suggest continuity over time, even while adult practices may be have a stronger association with adult outcomes. Results from this nationally representative sample of racially and ethnically diverse emerging adults supports the idea that race differences in abstinence from alcohol use are likely due, at least in part, to differences in religiosity that are informed by cultural expectations and social influences.

### 4.3. Limitations

Analysis of the NCS-R provides the opportunity to examine the potential impact of early adverse events on alcohol use and disorders, although there are limitations with the use of retrospective data. While clearly operationalized reports of adverse experiences during childhood tend to be reliable [80], some of the included items such as family welfare status may not be known to respondents, especially if it occurred when they were very young. Outcome variables rely on recall for age of onset and early regular use, which may have greater likelihood of inaccuracy for older respondents in the sample of emerging adults where more time has elapsed since incidents in question. This study includes full diagnostic information about alcohol disorders in a community sample of emerging adults, whereas most other large studies are limited to information regarding alcohol use patterns. On the other hand, the religiosity measures of the NCS-R are broad strokes relying on adult memories of childhood attitudes. This highlights the need for prospective studies on how families in adversity actively use both religious and spiritual resources and pass those values onto their children and the mechanisms by which faith communities can be protective in the face of adversity. In addition, the NCS-R lacks information on peer influence that is critical for fully understanding substance use patterns. Future analyses using the CPES can be used to explore similar questions religiosity and alcohol use for emerging adults. Study subsets of Latinos and Asians (NLAAS) and African Americans and Afro-Caribbeans (NSAL) lack some of the early adversity measures, the NSAL in particular contains more extensive and nuanced measures of religiosity and spirituality.

### 4.4. Implications

One protective mechanism of religiosity related to alcohol use problems may be abstention expectations for some denominations [66,72], and those may moderate alcohol use for college students in the context of norms among peers that include high frequency and quantity of drinking [81]. Research also suggests that social integration and community membership provide important social support, apart from the normative context of a particular denomination [82]. Further investigation of both of these theoretical approaches should take into account gender roles and expectations, as well as cultural differences by racial/ethnic group, and acculturation for immigrant youth. Evidence of potential protective factors of religiosity can be used to consider how programming related to risk can be integrated into church youth programs and in pastoral care settings. Mental health care might also consider how to be more inclusive of religiosity and spirituality (in addition to/beyond 12 step) in treatment settings, if clients indicate a desire to integrate these beliefs and practices into their care.

## Figures and Tables

**Table 1:** Descriptive statistics for key variables by gender.

Weighted	Full Sample %	Male %	Female %	Chisq *p*
*Alcohol Use and Disorder*				
Early First Drink	52.56	53.61	51.54	0.6306
Early Regular Drinker	21.77	24.70	18.84	0.0352
Any Alcohol Disorder	14.49	19.38	9.69	0.0004
*Religiosity and Spirituality*				
Importance of Religion as a Child (very or somewhat important)	74.06	70.05	77.99	0.0338
Church Attendance as an Adult (once a month or more)	42.87	33.30	52.27	0.0009
Religious Beliefs Guide Decisions (often or sometimes)	63.45	56.94	69.52	0.0184

Notes: Italicized titles in the tables indicate conceptually-based constructs which are also entered as blocks in the regression analyses.

**Table 2:** Descriptive statistics for key variables by race.

Weighted	Full Sample %	Asian %	Black %	Latino %	White %	Chisq *p*
*Alcohol Use and Disorder*						
Early First Drink	52.56	43.40	47.63	54.03	51.99	0.3051
Early Regular Drinker	21.77	6.34	8.86	18.25	21.69	0.0520
Any Alcohol Disorder	14.49	5.40	4.26	14.89	13.86	0.2028
*Religiosity and Spirituality*						
Importance of Religion as a Child (very or somewhat important)	74.06	85.81	82.65	84.55	73.23	0.0531
Church Attendance as an Adult (once a month or more)	42.87	61.63	52.15	45.59	42.14	0.4979
Religious Beliefs Guide Decisions (often or sometimes)	63.45	78.28	83.45	67.82	62.24	0.0169

Notes: Italicized titles in the tables indicate conceptually-based constructs which are also entered as blocks in the regression analyses.

**Table 3:** Final logistic regression models for (**Model A**) early onset of alcohol use; (**Model B**) early regular alcohol use patterns; and (**Model C**) alcohol abuse or dependence (*n* = 900).

Weighted	Model A Early Onset	Model B Early Regular Drinker	Model C Abuse or Dependence
Gender (male = 1)	1.12 [0.78,1.60]	1.61 ** [1.13,2.30]	1.27 [0.58,2.76]
*Race/Ethnicity*			
Asian	0.85 [0.21,3.43]	0.29 * [0.10,0.87]	0.60 [0.09,4.15]
Black	0.69 [0.35,1.33]	0.27 ** [0.11,0.67]	0.32 * [0.13,0.80]
Latino	1.28 [0.63,2.61]	0.85 [0.44,1.66]	0.95 [0.45,1.98]
White	1	1	1
Maternal Education Level (in years)	1.07 [0.99,1.15]	1.03 [0.94,1.12]	0.91 [0.77,1.08]
*Childhood Adversity*			
Family on welfare 6 months+	1.48 [0.80,2.76]	1.25 [0.67,2.33]	1.58 [0.63,4.00]
Mom w/substance use	1.40 [0.66,2.97]	2.15 * [1.01,4.59]	2.87 ** [1.52,5.44]
Dad w/substance use	1.89 * [1.14,3.11]	1.68 [0.98,2.88]	2.27 *** [1.40,3.67]
Mother had periods of sadness 2+ weeks	1.79 ** [1.22,2.63]	1.95 ** [1.30,2.93]	0.81 [0.43,1.52]
Unsupervised at too early age (recoded to dichotomous)	1.47 [0.54,4.03]	3.53 ** [1.40,8.88]	0.41 * [0.18,0.90]
Hungry/parents did not fix meals (recoded to dichotomous; 17 cases)	0.96 [0.40,2.31]	0.13** [0.03,0.58]	0.97 [0.34,2.79]
*Childhood Protective Factor*			
Lived with both biological parents until age 16	0.54 ** [0.34,0.86]	0.69 [0.43,1.10]	1.27 [0.79,2.04]
*Religiosity*			
Importance of Religion as a Child	0.56 * [0.32,0.98]	0.80 [0.48,1.35]	0.58 [0.22,1.52]
Frequency of Church Attendance as Adult			0.21 *** [0.10,0.45]
Religious Beliefs Guide Decisions			2.19 [0.86,5.59]
Early Regular Drinker			7.40 *** [4.41,12.39]
Importance of Religion as a Child × Male			2.53 * [0.98,6.53]
Frequency of Church Attendance as Adult × Male			4.89 ** [1.84,13.03]
Religious Beliefs Guide Decisions × Male			0.36 [0.11,1.12]

Notes:

* *p* < 0.05;

** *p* < 0.01;

*** *p* < 0.001; Italicized titles in the tables indicate conceptually-based constructs which are also entered as blocks in the regression analyses.
